# The impact of video performance technology and peer-to-peer learning on table tennis skill acquisition in elementary students

**DOI:** 10.3389/fspor.2025.1653334

**Published:** 2025-10-01

**Authors:** Di Feng, Victor R. A. Cossich, Emad Abdelrasoul, Alexandre Monte Campelo, Larry Katz

**Affiliations:** ^1^ Department of Physical Education, Harbin Engineering University, Harbin, China; ^2^ Faculty of Kinesiology, University of Calgary, Calgary, AB, Canada; ^3^ Faculty of Physical Education, Benha University, Benha, Egypt

**Keywords:** peer learning, physical education, video feedback, technology integration, table tennis skills, motor skill acquisition

## Abstract

**Introduction:**

Peer-to-peer (P2P) learning promotes collaboration, critical thinking, and active student participation, with recognized benefits in classroom settings. However, its integration into physical education (PE), particularly in combination with video performance technology—tools for learners to record and evaluate motor skills through structured video feedback—remains underexplored. Multimedia tools like video feedback have shown promise in enhancing motor skill acquisition, but their effectiveness in PE environments is not yet fully understood. Thus, we aimed to evaluate the impact of combining video performance technology with P2P learning on the table tennis skills.

**Methods:**

A quasi-randomized control trial was conducted with 73 Grade 6 students from four PE classes. Participants were divided into four groups: Instruction Sheets Group, iPad Camera Group, Instructed Video Group (using the Move Improve® app), and a Traditional Learning Group. Over 2 weeks, all groups completed seven 45-minute table tennis sessions focusing on grip, stance, forehand, and backhand strokes. Pre- and post-assessments were conducted, and a mixed-design ANOVA was used to evaluate performance improvements across groups.

**Results:**

All groups demonstrated significant skill improvements. The Instructed Video Group and Traditional Learning Group showed the greatest skill improvements. The Move Improve® app, which provides structured video demonstrations and guided peer feedback, helped students effectively analyze and refine their movements.

**Conclusion:**

Integrating video technology with P2P learning can match the effectiveness of expert-led instruction and provides additional benefits such as improved engagement and self-assessment. These findings support the broader use of multimedia tools to enhance skill development in PE, especially where expert instruction is limited.

## 1 Introduction

Peer-to-peer learning (P2P) is an educational practice in which students interact with other students to attain educational goals (1–3). Students enhance their learning by explaining their ideas to peers and participating in structured activities designed to promote mutual knowledge exchange (1, 4). They develop skills in organizing and planning learning activities, working collaboratively, giving and receiving feedback, and evaluating their own learning (5, 6).

As a potential outcome of P2P interaction, a cognitive conflict can arise, stemming from feedback and peers' diverse beliefs and perspectives. When exposed to differing perspectives during peer interactions, learners may experience cognitive conflict, which encourages them to reconsider their prior knowledge, incorporate new information, and refine their understanding of the content (7, 8). This necessitates a shift in paradigm from highly teacher-centred to learner-centred education, in which students are expected to take greater initiative and responsibility to manage more of their own learning and educational/personal development (9, 10). Studies in general education settings have shown that peer learning enhances active participation, motivation, and learning attitudes (10–12). It has also been shown that P2P reduces the teacher's burden—an important factor in large class settings where individualized feedback is challenging—and enhances learners' reflective ability and engagement (13, 14).

However, P2P learning approaches seem to have gained less attention concerning physical education (PE) (5, 15–17), especially when dealing with children and young people (18–20). There is a great need to further study the effectiveness of using technology in PE classes, particularly because many PE settings face challenges such as large class sizes, limited time for individual instruction, and varying levels of teacher expertise. Technology can help address these challenges by enhancing student engagement, enabling more frequent feedback, and supporting peer and self-assessment.

Nowadays, information technology provides students with excellent opportunities to learn (3). Also, digital technologies have been reported to increase students' motivation (21) and engagement (6), cognitive understanding (22), support assessment (23) and assist in learning and performing motor skills (24). Moreover, studies have demonstrated that integrating technology with P2P in PE classes promotes the students' learning interest, motivation, and improves skill execution, as it facilitates the correction of errors in skill performance (25–27). However, some studies have reported no significant improvements in students' skill performance following technology-based interventions in PE. For instance, Papastergiou et al. (28) found that a blogging-based multimedia intervention did not enhance basketball skills, likely due to reduced active practice time and the reflective—rather than performance-focused—nature of the task. Similarly, Niźnikowski et al. (29) reported limited gains, potentially due to insufficient integration of the technology with skill-specific instruction.

This mix of observations suggests that selecting a well-defined pedagogical approach to support technology use will not automatically result in positive learning experiences (62). Consequently, it is unclear why students' learning outcomes vary and how they respond to integrating technology with peer learning. These variations may stem from individual differences in cognitive processing and prior experience with multimedia environments (30). As such, despite growing interest in technology-enhanced peer learning, there is still a lack of research in PE settings, particularly concerning motor skill development in children. Given the specific instructional challenges of PE, this integration remains underexplored. Therefore, there is a significant need to examine the effectiveness of combining technology with P2P approaches in PE.

Learning from multimedia (e.g., video, photo, audio, text) is widely recognized as beneficial for providing students with control over their learning process, allowing students to freely navigate through learning content (30, 31). It is not a surprise that there has been a noticeable surge in the use of multimedia for motor skills learning (17, 32–35). Due to the strong appeal of screen-based technologies among children (36), integrating them into PE can create more engaging lesson plans (22). Especially the visual feedback provided through video performance technology allows children to observe and compare their own movements with both model and peer performances. This supports technical understanding and facilitates self-correction. As a result, children develop greater self-efficacy and perceived social support, as the feedback becomes more concrete, personalized, and engaging (37). In this direction, our group has created a user-friendly performance analysis tool designed to improve an individual's ability to perform a skill (Move Improve®—MI). MI is unique because it does not require users to have prior expertise in motor skill evaluation. The app provides intuitive, step-by-step guidance using demonstration videos and simple component-based questions, enabling both peers and performers to engage in the assessment process regardless of previous experience. It allows users to compare their performance to a standard demonstration video, breaking down physical skills into easily comprehended components supported by multimedia content in a P2P learning approach. For instance, when learning a basic motor skill such as an overhand throw or a jumping technique, MI helps students analyze each phase of the movement—such as preparation, execution, and follow-through—using segmented video clips and simple visual cues to support peer feedback and self-assessment. Unlike simply watching an instructional video, video performance technology provides an interactive process in which learners actively analyze their own performance, segment skills, and exchange peer feedback. This active engagement distinguishes it from passive observation and has been shown to be more effective in supporting motor skill learning (37, 38).

Previous studies have demonstrated that using MI facilitated improved skill performance among practitioners through P2P learning (39, 40). However, this technology has never been tested for teaching table tennis. Table tennis is a complex sport that demands fine motor coordination, quick decision-making, and precise timing, making it an ideal setting to evaluate the potential of multimedia-based peer learning strategies. Although research on the use of technology in table tennis is limited, some studies have examined video-based feedback and performance analysis in this sport, indicating its potential to support technical learning (41, 42). However, no studies to date have combined video performance technology with P2P learning in table tennis. It is expected that multimedia P2P interventions will demonstrate advantages over traditional PE methods, which typically follow a “mimic/practice” approach where the teacher demonstrates the skill with minimal feedback (43). This resultant learning occurs because visual feedback enables students to compare their own movements with model and peer performances, identify errors, and make immediate adjustments. In addition, exchanging feedback reinforces metacognitive engagement and self-efficacy, which supports motor skill acquisition (30, 37).

This study aims to investigate the effectiveness of integrating video performance technology with P2P learning in enhancing students' skill execution in PE classes, compared to traditional learning methods. Specifically, we hypothesize that the integration of video performance technology, such as MI, with a P2P learning approach, will lead to significantly greater improvements in students' skill execution than those observed with traditional learning methods.

## 2 Methods

### 2.1 Research design

This was a quasi-randomized control trial study aimed at investigating the efficacy of different pedagogical interventions within the context of Table Tennis Classes, assessing their impact on skill enhancement in traditional and P2P learning settings. The experiment extended over 2 weeks, with three sessions in the first week and four sessions in the second week, comprising seven sessions in total, each lasting approximately 45 min. In the first and last sessions, students of all groups were required to complete the skills test for grip, stance, forehand stroke, and backhand stroke. The design encompassed pre- and post-test sessions for skill assessment. This comprehensive methodology allowed for a thorough exploration of the effects of the interventions on both skill acquisition and perceptual changes among participants. The structure of this intervention builds upon previous studies that have examined technology-supported learning in PE (28, 29). Moreover, research using the MI tool in other contexts has employed similar peer-based designs to evaluate skill development (39, 40). Studies in table tennis have also explored video-based feedback approaches to support technical learning (41, 42). Our study extends this work by combining video performance technology with P2P learning in table tennis, a context not previously explored. No attrition occurred during the study, and all participants completed both pre- and post-test assessments.

### 2.2 Study participants

A total of 73 elementary school students (36 boys and 37 girls, mean age = 10.9 ± 0.4 years) from four Grade 6 classes in an elementary school in Calgary, Canada, participated in the study. The four classes were randomly assigned to one of four pedagogical interventions. All students in each class participated in the activities, but only the results from those students who assented and whose parents signed the consent forms were included in the findings. Participants in the Instruction Sheets Group (ISG, *n* = 18) utilized instruction sheets for both learning and evaluation during peer interactions. Students in the iPad Camera Group (CG, *n* = 18) utilized iPad cameras for both learning and peer evaluation. Participants in the Instructed Video Group (IVG, *n* = 18) utilized the MI for iPad for both learning and evaluation purposes. Lastly, students in the Traditional Learning Group (TG, *n* = 19) traditionally received instruction and evaluation by the instructor. Gender distribution within each group was balanced, with ISG (M = 9, F = 9), CG (M = 9, F = 9), IVG (M = 9, F = 9), and TG (M = 9, F = 10). The study obtained approval from the local ethics committee. None of the participants had prior formal experience with table tennis, and therefore they can be considered novice learners.

### 2.3 Move improve mobile application

**Figure 1:**
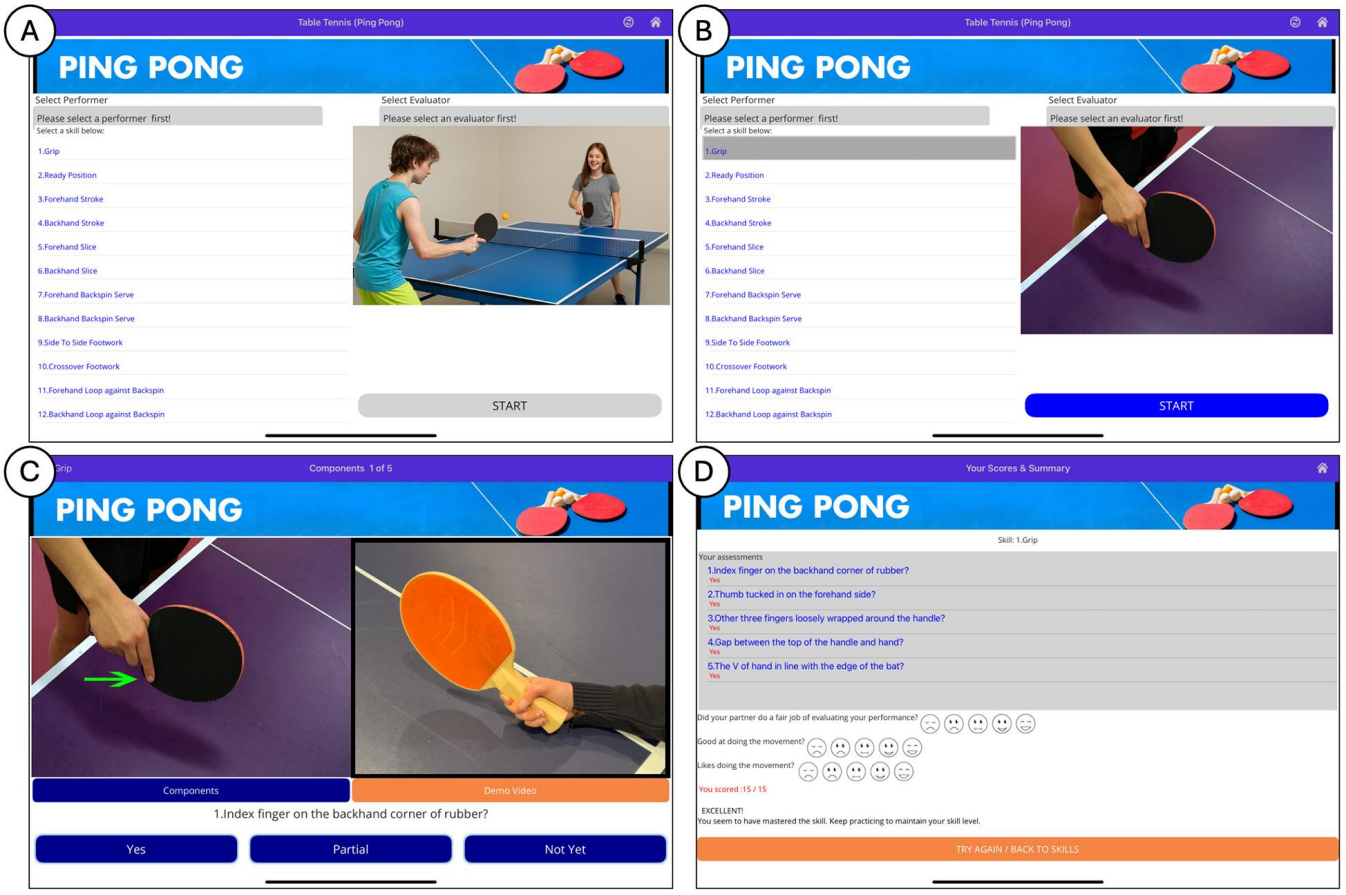
Interface of the Move Improve® application for peer-to-peer skill evaluation in table tennis. Screenshots of the Move Improve® interface during a peer-to-peer (P2P) table tennis evaluation session. **(A)** Skill selection screen where students choose the performer and evaluator roles and select a specific skill to assess. **(B)** Example of the setup screen for evaluating the “Grip” skill, with a visual reference image of the movement. **(C)** Component evaluation screen showing the video-based prompt and rating options (“Yes”, “Partial”, “Not Yet”) for a specific skill subcomponent (e.g., finger placement). **(D)** Summary screen showing peer scores across multiple components, self-reflection items (e.g., partner fairness, self-performance, enjoyment), and feedback encouraging continued practice. This structured, step-by-step interface supports P2P learning through multimedia cues and guided assessments of key skill components.

The MI mobile application is a versatile tool designed to assess and enhance movement skills. Users have the flexibility to evaluate their own performance or that of their peers across a range of predefined skills. Upon selecting a skill from the pre-created list within the MI app, users are presented with an instructional video demonstrating the proper execution of the skill. This video serves as a reference guide, providing users with visual guidance on technique and form. Following the instructional video, users are guided through a component list detailing sequential movements and key points necessary for skill mastery. During peer assessment, one individual assumes the role of evaluator while the other acts as the performer. The evaluator records a video of the performer executing the skill using the MI app, which is then reviewed collaboratively. The MI app facilitates a systematic assessment process, allowing evaluators to analyze each skill component individually. Evaluators provide ratings based on the accuracy of the performer's execution, with options including “Yes” for correct performance (score = 3), “Partial” for incomplete execution (score = 2), and “Not Yet” for incorrect performance (score = 1). Upon completion of the assessment, a summary list of components and their corresponding scores is generated within the MI app, providing evaluators and performers with tangible feedback. This feedback serves to identify areas of strength and areas for improvement, guiding future practice sessions. The collaborative nature of the assessment process encourages open dialogue between evaluators and performers, fostering a supportive learning environment. Additionally, evaluators and performers are encouraged to switch roles and conduct a new assessment, further promoting mutual learning and skill development. Overall, the MI serves as a valuable tool for skill assessment and enhancement, offering users a structured approach to skill evaluation and personalized feedback (Figure 1).

### 2.4 Table tennis classes

A highly experienced professional table tennis instructor, with 10 years of teaching experience, worked with the students to teach four essential skills: Grip, Stance, Forehand Stroke, and Backhand Stroke. Briefly, the grip refers to how a player holds the racket, influencing control and power. A stable and balanced stance is essential for mobility and agility, facilitating quick movements to reach the ball effectively. The forehand stroke involves swinging the racket forward to make contact with the ball on the forehand side, aiming for power, spin, and accuracy. Conversely, the backhand stroke involves swinging the racket backward to make contact with the ball on the backhand side, requiring proper technique for power, spin, and control. Throughout the experiment, students were trained to utilize various types of learning media, including instruction sheets, cameras, and instructed videos, for peer learning and evaluation, according to group allocation. In the second and third sessions, students focused on learning and reviewing grip, stance, and forehand strokes. Subsequently, the fourth and fifth sessions were dedicated to the instruction and review of the backhand stroke. Finally, in the sixth session, a comprehensive review of both forehand and backhand strokes was conducted. All classes followed a standardized structure, which included warm-up exercises, initial verbal instruction by the teacher, practice sessions, evaluation (conducted by peers or the teacher), and a class summary. This consistent structure ensured uniformity across all sessions and provided students with a familiar routine conducive to learning and skill development.

**Figure 2:**
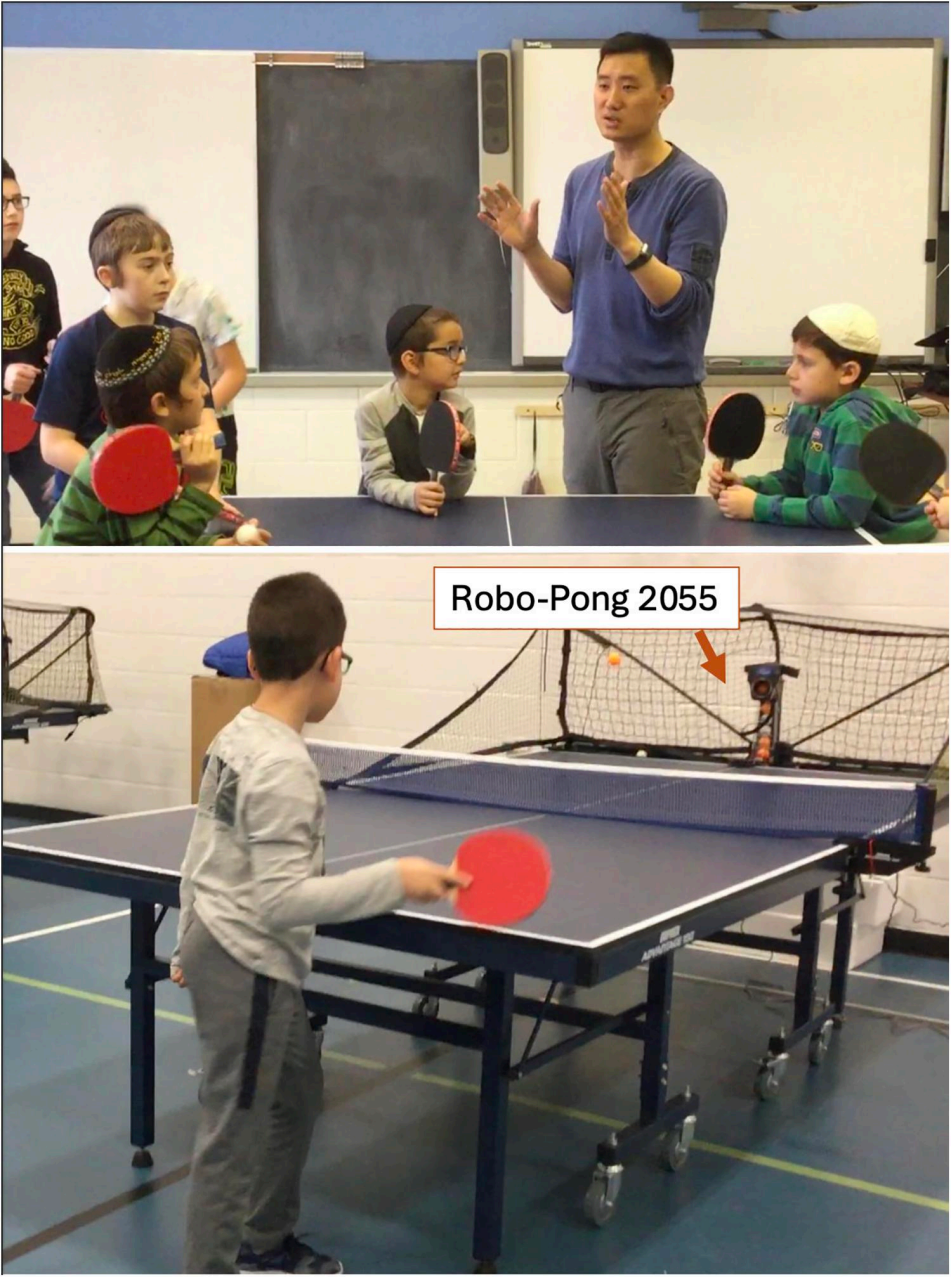
Example of a table tennis lesson. Top: A table tennis instructor provides verbal instruction and demonstration to a group of elementary school students before initiating skill practice. Bottom: A student engages in a forehand stroke task using a *Robo-Pong 2055* ball-feeding machine, which delivers consistent ball trajectories to support individualized repetition. The robot-enabled setup facilitates deliberate practice and immediate feedback opportunities during peer-assisted and teacher-guided learning.

Training partners were randomly assigned, and students maintained the same partners throughout the seven training sessions. Students in the ISG utilized instruction sheets for learning and evaluation of peers. These sheets included the same skill components presented in the MI app, but without visual images or scaffolds (Table 1). The CG utilized iPad cameras for both learning and peer evaluation. Participants in IVG utilized the MI iPad application for both learning and evaluation purposes. Lastly, students in the TG were taught traditionally—a teacher-centered approach where the instructor demonstrates the skill and students practice—without extra media, for both learning and evaluation. All students were given the same practice time, which was limited by the class duration. The same checklist of critical elements was used for all groups, ensuring standardization across conditions. These elements were derived from the instructional sheet and mirrored exactly those used in the MI app. Each skill was evaluated using the same predefined set of performance criteria. Regardless of the approach used, performance analysis was conducted based on the same components (Table 1) and scoring system (“Yes” = 3, “Partial” = 2, and “Not Yet” = 1). The total points for each skill determined the performance score. To facilitate consistent ball placement during practice sessions, two ping-pong robot machines (Robo-Pong 2055, Newgy Industries, Hendersonville, USA) were employed across all four experimental groups. These machines served balls to predetermined locations on the ping-pong tables, ensuring standardized conditions for all participants (Figure 2).

**Table 1:** Table tennis performance analysis system.

Grip (maximum points 15)	1. Index finger on the backhand corner of rubber 2. Thumb tucked in on the forehand side 3. Other three fingers loosely wrapped around the handle 4. Gap between the top of the handle and hand 5. The V of hand in line with the edge of the bat
Stance (maximum points 24)	1. Feet slightly wider than shoulder width apart 2. The dominant foot slightly behind the non-dominant side 3. Knees slightly bent 4. Body leaning forward 5. Both arms out in front of body 6. Elbow bent about 90–110 degrees 7. Standing about an arm's length away from the table 8. Weight distributed between the balls of both feet
Forehand stroke (maximum points 30)	1. Body rotates from hips towards the back foot 2. Elbow and bat rotate with the body, and bat angle closes 3. Body initiates the movement of the arm 4. Hips and shoulders rotate towards the ball 5. Arm moves forward with the body 6. Bat angle stays closed throughout the shot 7. Contact the ball at the peak of the bounce and out in front of you 8. Keep a fist-length gap between the elbow and the body 9. Follow through, forward and upward, after contacting the ball 10. Return to the ready position
Backhand stroke (maximum points 30)	1. Bring the bat backwards and down just in front of belly button 2. The backhand rubber of the bat points in the direction you wish to play 3. Arm moves forward and slightly up 4. The movement comes from the elbow and forearm 5. Create a slightly closed bat angle, and the bat angle stays closed throughout the shot 6. Contact the ball at the peak of the bounce and out in front of you 7. Keep a fist-length gap between the elbow and the body 8. Follow through, forward and upward 9. Bat finishes at chin level, pointing where you have hit the ball 10. Return to the ready position

Evaluators provide ratings based on the accuracy of the performer's execution, with options including “Yes” for correct performance (score = 3), “Partial” for incomplete execution (score = 2), and “No” for incorrect performance (score = 1).

### 2.5 Statistical analysis

A mixed-design ANOVA was conducted for each skill (grip, stance, forehand stroke, and backhand stroke), with time (first vs. last session) as the within-subjects factor and group (ISG, CG, IVG, TG) as the between-subjects factor. This omnibus model tested main effects of time and group, as well as their interaction. Within-group comparisons (pre vs. post) were additionally performed using paired *t*-tests; when normality of the difference scores was not met, the Wilcoxon signed-rank test was applied. For each contrast, we reported the mean change (Δ), its 95% bootstrap confidence interval, and the effect size (Cohen's *d* for parametric tests, or rank-biserial correlation for Wilcoxon). *P*-values were adjusted for multiple comparisons within each skill using Holm's method. Between-group comparisons of change were assessed using a one-way ANOVA on the difference scores (Δ = post—pre), followed by Bonferroni-adjusted pairwise comparisons. Hedges' *g* was reported as the effect size for between-group contrasts. For descriptive purposes, percentage change (Δ%) from pre to post was also summarized by group. For all analyses, the alpha level was set at 5%. The statistical analyses were implemented using Pingouin (version 0.5.4), and the graphics were generated with Matplotlib for Python (version 3).

## 3 Results

Across the intervention, all groups improved on every skill, as shown by a consistent main effect of time (*p* < 0.001, for all skills). Change-score analyses indicated differential gains for Grip and Forehand, with IVG and TG outperforming ISG/CG for Grip and TG improving more than CG for Forehand. In contrast, Stance and Backhand showed comparable pre–post improvements across groups. Consistent with these patterns, the mixed ANOVA interaction was significant for Forehand only, while Stance and Backhand displayed strong time effects without reliable Group × Time interactions. Detailed skill-specific results follow.

### 3.1 Grip

**Figure 3:**
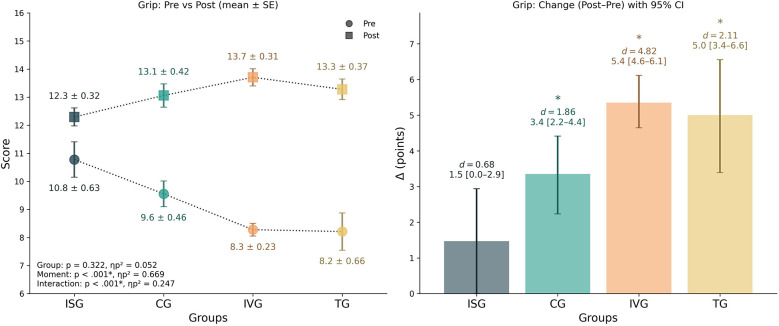
Grip performance by group. Left panel: group means (±SE) at pre and post. Right panel: change scores (Δ = post–pre) with 95% bootstrap CIs (5,000 resamples). The inset in left panel reports mixed-design ANOVA results (*p* and *ηp*^2^) for Group, Time, and Group × Time. * Indicate significant within-group pre–post comparisons (*p* < 0.05). *d* denotes the paired-samples effect size (Cohen's *d*). *Post-hoc* on change scores showed IVG > ISG (*p* < 0.001), IVG > CG (*p* = 0.043), and TG > ISG (*p* = 0.021); other contrasts were not significant. Positive Δ indicates improvement.

The mixed-design ANOVA revealed no significant main effect of group [*F*_(3,63)_ = 0.39, *p* = 0.760, *ηp*^2^ = 0.018], but a strong main effect of time [*F*_(1,63)_ = 8,510.97, *p* < 0.001, *ηp*^2^ = 0.993], with no significant interaction between group and time [*F*_(3,63)_ = 1.84, *p* = 0.097, *ηp*^2^ = 0.080]. Within-group analyses showed that the CG, IVG, and TG groups demonstrated significant improvements from pre- to post-test (Δ = 3.35 [95% CI 2.24–4.41], *d* = 1.86, *p* < 0.001; Δ = 5.35 [95% CI 4.65–6.12], *d* = 4.82, *p* < 0.001; Δ = 5.00 [95% CI 3.39–6.56], *d* = 2.11, *p* < 0.001, respectively). The ISG group showed a smaller, non-significant gain [Δ = 1.47 (95% CI 0.00–2.94), *d* = 0.68, *p* = 0.075]. Between-group analysis of the change scores indicated a significant group effect [*F*_(3,65)_ = 7.10, *p* < 0.001, *ηp*^2^ = 0.247]. *Post hoc* tests revealed that the IVG group improved more than ISG and CG (*p* < 0.001 and *p* = 0.043, respectively), and TG improved more than ISG (*p* = 0.021). No other between-group contrasts reached significance. Descriptively, IVG and TG exhibited the largest relative percentage improvements (66% and 81%, respectively), whereas ISG showed a smaller average gain (22%). Overall, these findings indicate that all groups improved their Grip performance across the intervention, but the gains were particularly pronounced in the IVG and TG groups (Figure 3).

### 3.2 Stance

**Figure 4:**
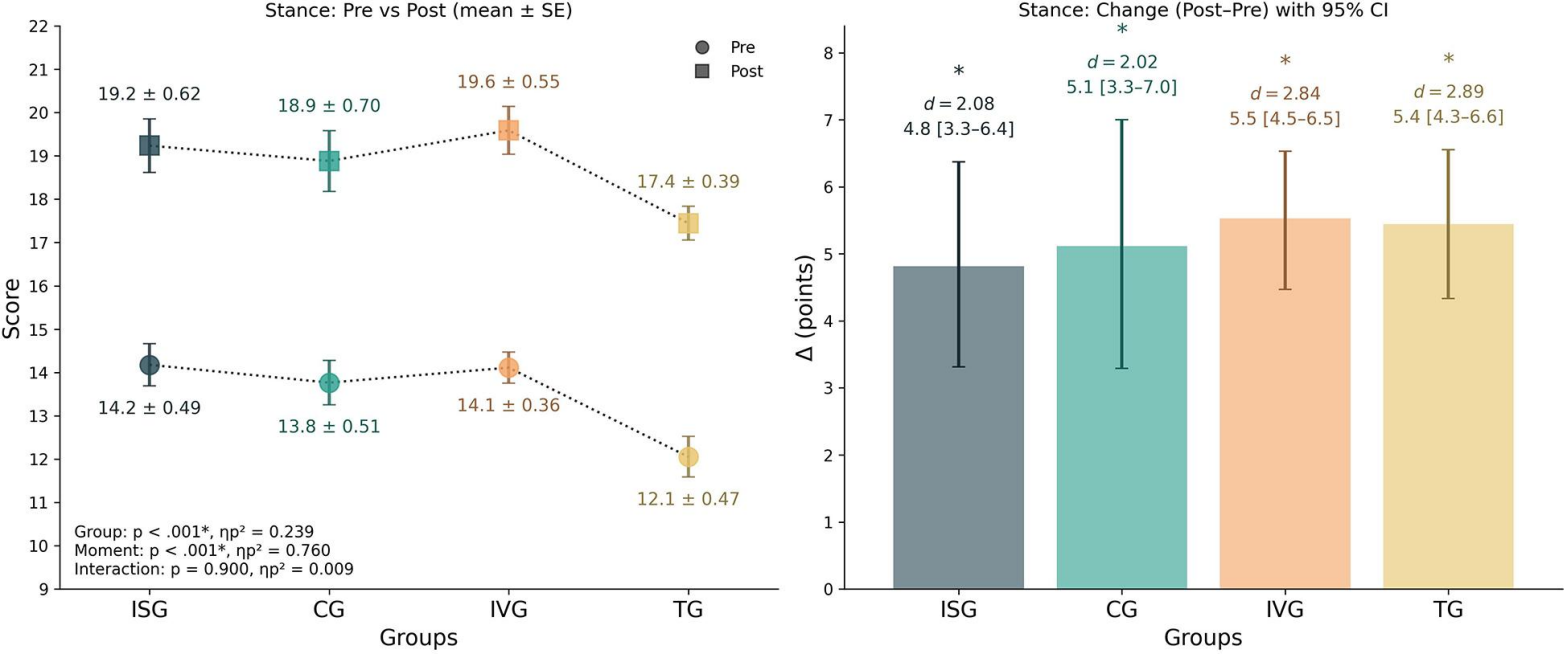
Stance performance by group. Left panel: group means (±SE) at pre and post. Right panel: change scores (Δ = post–pre) with 95% bootstrap CIs (5,000 resamples). The inset in left panel reports mixed-design ANOVA results (*p* and *ηp*^2^) for Group, Time, and Group × Time. * Indicate significant within-group pre–post comparisons (*p* < 0.05). *d* denotes the paired-samples effect size (Cohen's *d*). *Post-hoc* on change scores showed no significant between-group differences after Bonferroni correction (all *p* = 1.0). Positive Δ indicates improvement.

The mixed ANOVA revealed a significant main effect of group [*F*_(3,64)_ = 6.69, *p* = 0.001, *ηp*^2^ = 0.239] and a strong main effect of time [*F*_(1,64)_ = 202.55, *p* < 0.001, *ηp*^2^ = 0.760], while the interaction between group and time was not significant [*F*_(3,64)_ = 0.19, *p* = 0.900, *ηp*^2^ = 0.009]. Within-group analyses showed significant pre–post improvements in all groups: ISG improved by +4.81 points [95% CI 3.31–6.38], *d* = 2.08, *p* < 0.001; CG by +5.12 points [95% CI 3.29–7.00], *d* = 2.02, *p* < 0.001; IVG by +5.53 points [95% CI 4.47–6.53], *d* = 2.84, *p* < 0.001; and TG by +5.44 points [95% CI 4.33–6.56], *d* = 2.89, *p* < 0.001. The one-way ANOVA on the change scores (Δ) did not reveal significant between-group differences [*F*_(3,64)_ = 0.19, *p* = 0.900, *ηp*^2^ = 0.009], and pairwise *post hoc* comparisons confirmed no reliable contrasts (all *p* = 1.0). Descriptively, TG showed the largest relative percentage improvement (+49%), followed by IVG (+41%), CG (+41%), and ISG (+36%). Together, these findings indicate that stance performance improved markedly across all groups, with comparable magnitudes of change between them (Figure 4).

### 3.3 Forehand stroke

**Figure 5:**
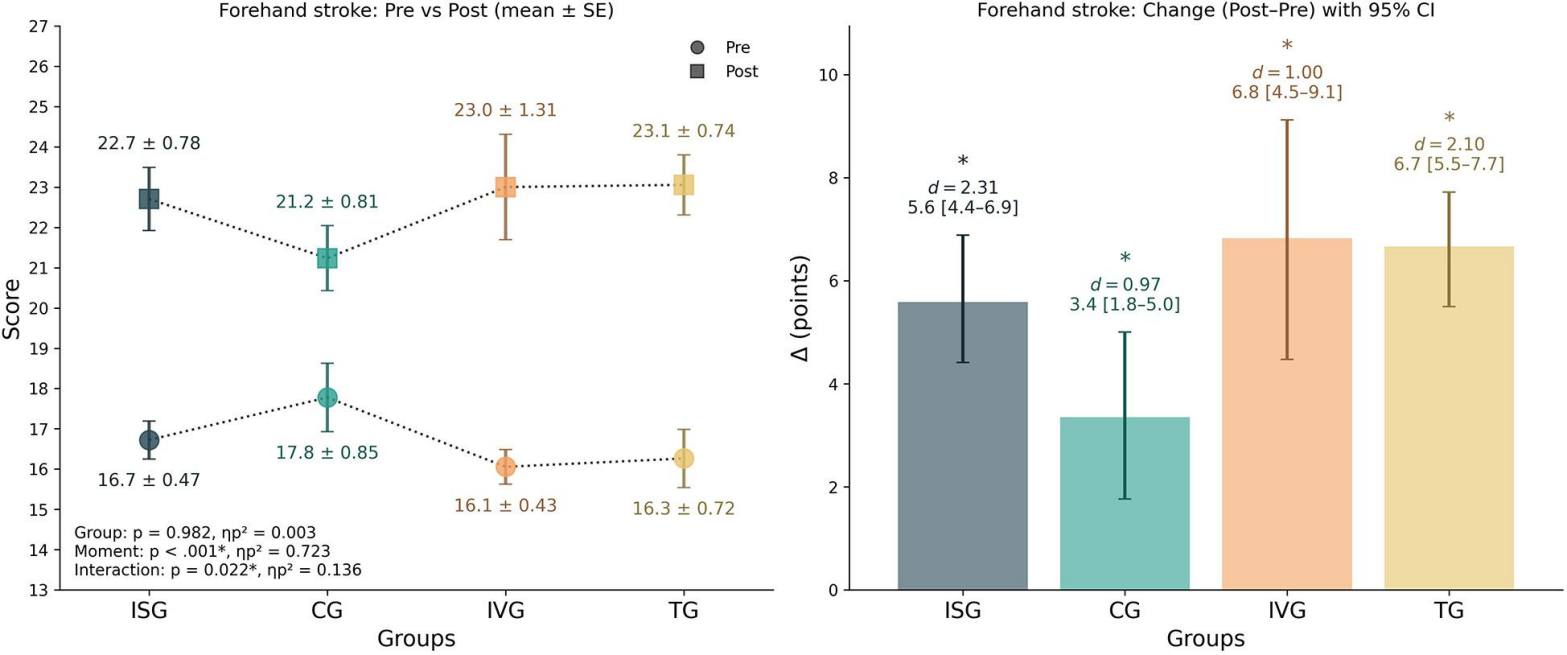
Forehand stroke performance by group. Left panel: group means (±SE) at pre and post. Right panel: change scores (Δ = post–pre) with 95% bootstrap CIs (5,000 resamples). The inset in left panel reports mixed-design ANOVA results (*p* and *ηp*^2^) for Group, Time, and Group × Time. * Indicate significant within-group pre–post comparisons (*p* < 0.05). *d* denotes the paired-samples effect size (Cohen's *d*). *Post-hoc* on change scores showed TG > CG (*p* = 0.017); other contrasts were not significant. Positive Δ indicates improvement.

The mixed-design ANOVA revealed no significant main effect of group [*F*_(3,65)_ = 0.06, *p* = 0.982, *ηp*^2^ = 0.003], but a robust main effect of time [*F*_(1,65)_ = 169.95, *p* < 0.001, *ηp*^2^ = 0.723]. Importantly, the interaction between group and time was significant [*F*_(3,65)_ = 3.42, *p* = 0.022, *ηp*^2^ = 0.136], indicating differential improvements across groups. Within-group analyses confirmed significant pre–post gains in all groups. ISG improved by +5.59 points [95% CI 4.41–6.88], *d* = 2.31, *p* < 0.001; CG by +3.35 points [95% CI 1.76–5.00], *d* = 0.97, *p* = 0.002; IVG by +6.82 points [95% CI 4.47–9.12], *d* = 1.00, *p* < 0.001; and TG by +6.67 points [95% CI 5.50–7.72], *d* = 2.10, *p* < 0.001. Between-group analysis of the change scores (Δ) indicated a significant group effect [*F*_(3,65)_ = 3.42, *p* = 0.022, *ηp*^2^ = 0.136]. *Post hoc* tests showed that TG improved significantly more than CG (*p* = 0.017, Hedges' *g* = –1.08), while ISG also tended to outperform CG, though this contrast did not survive correction (*p* = 0.254). No other pairwise comparisons were significant. Descriptively, the largest relative percentage improvements were observed in IVG (+43%) and TG (+42%), followed by ISG (+32%) and CG (+21%). Taken together, these findings indicate that although all groups improved forehand stroke performance, the magnitude of improvement was greater in the TG and IVG groups compared to CG (Figure 5).

### 3.4 Backhand stroke

**Figure 6:**
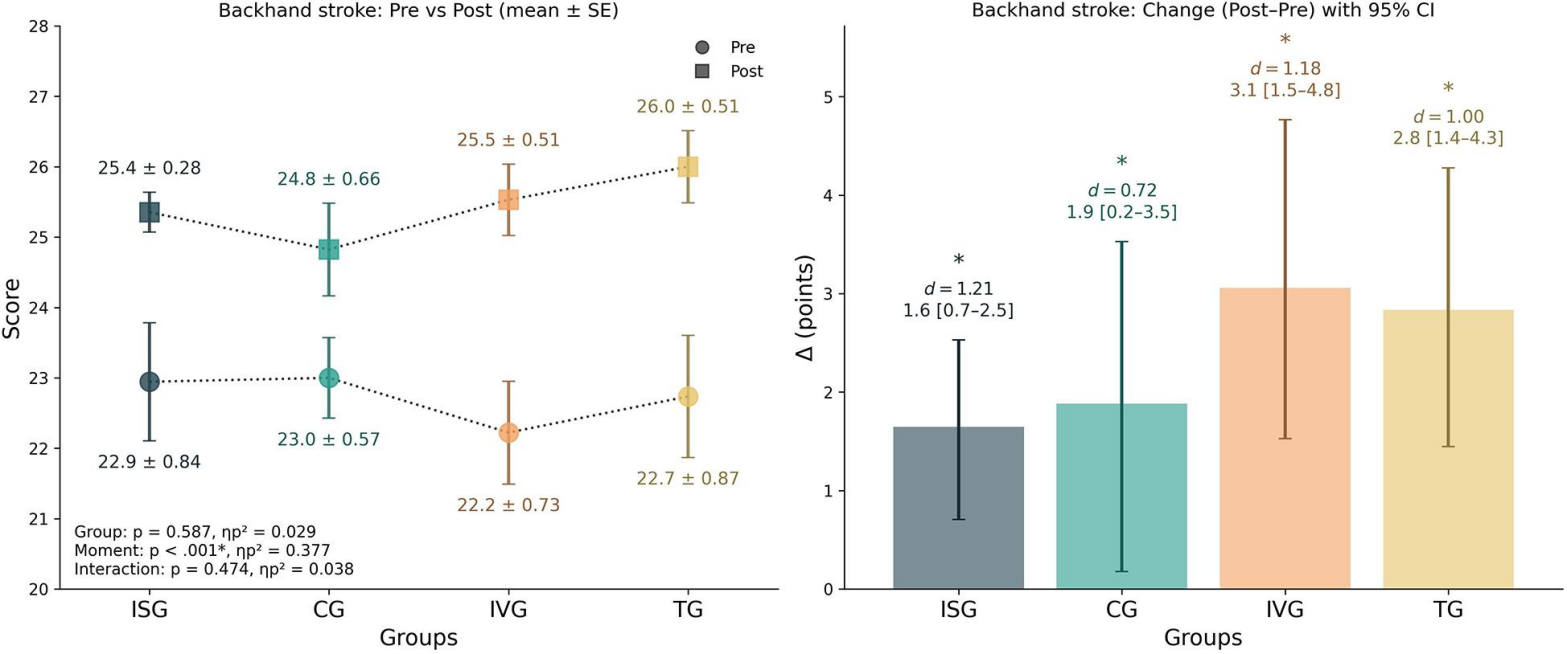
Backhand stroke performance by group. Left panel: group means (±SE) at pre and post. Right panel: change scores (Δ = post–pre) with 95% bootstrap CIs (5,000 resamples). The inset in left panel reports mixed-design ANOVA results (*p* and *ηp*^2^) for Group, Time, and Group × Time. * Indicate significant within-group pre–post comparisons (*p* < 0.05). *d* denotes the paired-samples effect size (Cohen's *d*). *Post-hoc* on change scores showed no significant between-group differences after Bonferroni correction (all *p* = 1.0). Positive Δ indicates improvement.

The mixed-design ANOVA revealed no significant main effect of group [*F*_(3,65)_ = 0.65, *p* = 0.587, *ηp*^2^ = 0.029], but a significant main effect of time [*F*_(1,65)_ = 39.29, *p* < 0.001, *ηp*^2^ = 0.377]. The interaction between group and time was not significant [*F*_(3,65)_ = 0.85, *p* = 0.474, *ηp*^2^ = 0.038], indicating that all groups improved to a similar extent. Within-group analyses confirmed significant pre–post improvements in all groups. ISG improved by +1.65 points [95% CI 0.71–2.53], *d* = 1.21, *p* = 0.008; CG by +1.88 points [95% CI 0.18–3.53], *d* = 0.72, *p* = 0.048; IVG by +3.06 points [95% CI 1.53–4.76], *d* = 1.18, *p* = 0.008; and TG by +2.83 points [95% CI 1.44–4.28], *d* = 1.00, *p* = 0.005. Between-group analysis of the change scores did not reveal significant group differences [*F*_(3,65)_ = 0.85, *p* = 0.474, *ηp*^2^ = 0.038], and all *post hoc* pairwise comparisons were non-significant after correction (all *p* = 1.000). Descriptively, IVG (+16%) and TG (+15%) showed slightly larger relative percentage improvements compared to ISG (+7%) and CG (+9%). In sum, all groups exhibited significant improvements in backhand stroke performance, with no evidence of differential gains between them (Figure 6).

## 4 Discussion

The present study examined the effectiveness of integrating video performance technology with P2P learning for enhancing students' motor skill execution in PE classes, compared to traditional instruction. All groups demonstrated significant improvements across the assessed skills (grip, stance, forehand stroke, and backhand stroke), confirming that structured practice and feedback are critical for skill development. Although the hypothesis that the IVG using the MI app would outperform the other groups was not fully supported, both the IVG and TG showed greater percentage improvements after the program. These findings indicate that multiple forms of feedback can effectively facilitate skill acquisition in table tennis, consistent with prior research demonstrating that feedback, regardless of modality, enhances learning by guiding attention, reinforcing correct performance, and supporting self-regulation (44–46).

Previous studies on P2P assisted by technology have demonstrated that skill performance can be improved (25, 39, 40, 47). These improvements may occur because feedback helps direct learners' attention to critical aspects of the skill, provides information for correcting errors, and supports self-regulation by enabling students to monitor and adjust their actions. In addition, technology-assisted environments can enhance these processes by offering clear, timely, and multimodal feedback that strengthens both understanding and retention (30, 45). However, introducing technology alone should not be considered the sole factor in facilitating the learning process (28), as elements such as student motivation, peer collaboration, the quality of instructional materials, and sufficient practice opportunities also play important roles.

Interestingly, the IVG demonstrated better performance compared to the other P2P learning groups in the present study (VG and CG). Considering that all these groups had access to the same evaluation sheet and metric method (Table 1), this suggests that the MI app used by IVG may offer features that enhance the evaluation and learning process, such as well-structured videos, skill snapshots with pictures of specific events during the movement, and one-by-one questions in the evaluation sheet to enhance the evaluator's concentration. Additionally, the immediate calculation and presentation of scores can expedite the exchange of information between peers, thus enhancing the learning process. This finding aligns with previous research suggesting that multimedia tools can enhance motor skill learning by offering visual and structured guidance (48). Good quality and timely feedback are key to supporting effective student learning, as it provides learners with specific information to improve performance and fosters self-regulation. In addition, constructive feedback contributes to building trust and dialogue between students and teachers, which strengthens the student–teacher relationship and creates a more supportive learning environment (49). Without frequent and accurate feedback, students may develop motor skills incorrectly, hindering skill acquisition and achievement (50).

The TG performance highlights the critical role of having a skilled and experienced tutor. The teacher in this study has more than 10 years of experience, which likely helped him develop effective internal methods of evaluation and ways to promote student change. This extensive experience contributes to the development of tacit knowledge—intuitive, hard-to-articulate knowledge gained through years of practice (51, 52). In the context of PE, tacit knowledge enables teachers to provide immediate, personalized feedback and adapt strategies to meet individual needs, fostering deeper skill understanding (53). The combination of expert guidance and the ability to tailor instruction to individual learners likely contributed significantly to the superior changes observed in the TG group.

A knowledgeable and experienced teacher can provide immediate, personalized feedback and adapt teaching strategies to meet the needs of individual students, fostering a deeper understanding of the skills being taught (54). The combination of expert guidance and the ability to tailor instruction to individual needs likely contributed significantly to the superior changes observed in the TG group.

An important practical consideration concerns instructional time allocation. In the peer-learning conditions, students devoted part of each session to guided observation, discussion, and peer evaluation, whereas the traditional group accumulated more uninterrupted ball-contact practice. Despite this difference in practice, the IVG achieved gains comparable to the TG, suggesting that structured video feedback and scaffolded peer analysis can deliver greater learning efficiency per unit of hands-on practice—consistent with models positing that targeted feedback and guided self/peer assessment focus attention on key performance elements and promote error correction (30, 37, 45, 46).

It is worth noting that some students expressed discomfort about being videotaped (IVG and CG). According to Ayres (55), people are accustomed to seeing their reflection in mirrors, which presents a reversed image. Since most faces are not perfectly symmetrical, the reflection differs from the true image seen on video, often creating a sense of unfamiliarity. This discrepancy can be unsettling as individuals search for evidence to fit their pre-existing mental models, reinforcing negative self-perceptions through confirmation bias (56). Moreover, feedback that aligns with existing self-concept tends to be more memorable and impactful (57). These mechanisms may explain the discomfort some students experienced when confronted with their own video recordings. Understanding these biases is crucial for educators using video technology in education, as supportive scaffolding is needed to help students acclimate and benefit from video-based feedback (58).

The study also highlights the importance of considering students' attitudes toward technology and peer learning. While the current results focus on skill improvement, future research should explore how these interventions impact students' motivation, engagement, and attitudes toward PE. These aspects are often conceptualized within models such as the Technology Acceptance Model (59), which emphasizes perceived usefulness and ease of use as drivers of technology adoption, or the Unified Theory of Acceptance and Use of Technology (60). Understanding these factors could provide deeper insights into how to best integrate technology and P2P learning in PE settings to maximize both skill acquisition and positive learning experiences (61).

Despite the promising findings, this study has some limitations that need to be considered. First, the sample size was relatively small and limited to a specific age group and geographic location, which may limit the generalizability of the results. Future studies should include a larger and more diverse sample to validate the findings. Second, the duration of the intervention was relatively short, spanning only 2 weeks. Longer-term studies are needed to assess the sustained impact of integrating video performance technology with P2P learning on skill acquisition and retention. Additionally, the randomization was by class rather than by students, so there may have been differences in classes that could have influenced the outcome. The study also did not account for potential confounding variables such as prior experience with technology or baseline skill levels, which could have influenced the outcomes. Moreover, no formal measures of students' engagement or self-assessment ability were collected, so these aspects could not be evaluated in the present study. While the MI app demonstrated benefits in this context, its effectiveness in other sports or educational settings remains to be tested. Future research should explore the applicability of such tools across different disciplines to fully understand their potential and limitations. Finally, future work should quantify and/or equate practice time across conditions (e.g., ball contacts, active practice minutes) and incorporate delayed retention tests; if the IVG's approach yields similar immediate gains with less hands-on practice, it may confer equal or superior retention via deeper processing (analysis, self-explanation, error diagnosis). Logging time-on-task (e.g., app usage logs or time-motion/session coding) alongside retention assessments will clarify whether peer/video analysis trades some immediate practice for higher retention of skills.

In conclusion, the integration of video performance technology with P2P learning in PE classes was found to be comparable in effectiveness to traditional instruction for improving students' table tennis skills. These findings suggest that innovative tools like the MI app can be applied as viable alternatives to traditional approaches in supporting motor skill development. Future studies should investigate whether such tools also influence students' motivation, engagement, and self-assessment, and examine their broader applicability across sports and educational contexts.

## Data Availability

The raw data supporting the conclusions of this article will be made available by the authors, without undue reservation.
